# Soluble Tie2 overrides the heightened invasion induced by anti-angiogenesis therapies in gliomas

**DOI:** 10.18632/oncotarget.7550

**Published:** 2016-02-21

**Authors:** Nahir Cortes-Santiago, Mohammad B. Hossain, Konrad Gabrusiewicz, Xuejun Fan, Joy Gumin, Frank C. Marini, Marta M. Alonso, Frederick Lang, W.K. Yung, Juan Fueyo, Candelaria Gomez-Manzano

**Affiliations:** ^1^ Department of Neuro-Oncology, The University of Texas MD Anderson Cancer Center, Houston, TX, USA; ^2^ Department of Neurosurgery, The University of Texas MD Anderson Cancer Center, Houston, TX, USA; ^3^ Comprehensive Cancer Center, Wake Forest University, Winston-Salem, NC, USA; ^4^ Department of Medical Oncology, University Hospital of Navarra, Pamplona, Spain; ^5^ Department of Genetics, The University of Texas MD Anderson Cancer Center, Houston, TX, USA; ^6^ Cancer Biology Program, The University of Texas Graduate School of Biomedical Sciences at Houston, Houston, TX, USA

**Keywords:** anti-angiogenesis, glioma, invasion, angiopoietin 2, Tie2-expressing monocytes

## Abstract

Glioblastoma recurrence after treatment with the anti–vascular endothelial growth factor (VEGF) agent bevacizumab is characterized by a highly infiltrative and malignant behavior that renders surgical excision and chemotherapy ineffective. Our group has previously reported that Tie2-expressing monocytes (TEMs) are aberrantly present at the tumor/normal brain interface after anti-VEGF therapies and their significant role in the invasive outgrowth of these tumors. Here, we aimed to further understand the mechanisms leading to this pro-invasive tumor microenvironment. Examination of a U87MG xenogeneic glioma model and a GL261 murine syngeneic model showed increased tumor expression of angiopoietin 2 (Ang2), a natural ligand of Tie2, after anti-angiogenesis therapies targeting VEGF or VEGF receptor (VEGFR), as assessed by immunohistochemical analysis, immunofluorescence analysis, and enzyme-linked immunosorbent assays of tumor lysates. Migration and gelatinolytic assays showed that Ang2 acts as both a chemoattractant of TEMs and an enhancing signal for their tumor-remodeling properties. Accordingly, *in vivo* transduction of Ang2 into intracranial gliomas increased recruitment of TEMs into the tumor. To reduce invasive tumor outgrowth after anti-angiogenesis therapy, we targeted the Ang-Tie2 axis using a Tie2 decoy receptor. Using syngeneic models, we observed that overexpression of soluble Tie2 within the tumor prevented the recruitment of TEMs to the tumor and the development of invasion after anti-angiogenesis treatment. Taken together, these data indicate an active role for the Ang2-Tie2 pathway in invasive glioma recurrence after anti-angiogenesis treatment and provide a rationale for testing the combined targeting of VEGF and Ang-Tie2 pathways in patients with glioblastoma.

## INTRODUCTION

Glioblastoma is the most common and aggressive type of primary brain tumor. Despite therapeutic advances over the past decade, the diagnosis of glioblastoma is associated with a median overall survival time of 15–18 months and a 5-year survival rate of less than 5% [1]. In addition, recurrence of glioblastoma after therapy is inevitable. Bevacizumab, an anti–vascular endothelial growth factor (VEGF) antibody, was approved in 2009 to treat glioblastoma recurrence on the basis of encouraging preclinical and clinical results [2, 3]. Although there is evidence that bevacizumab reduces tumor edema, angiogenesis, and disease burden, the use of this agent as well as other VEGFA/VEGFR2-targeting drugs has been followed by adaptive tumor responses in preclinical models and clinical settings [4–8]. Several anti-VEGF escape mechanisms are being explored including the induction of a heightened invasive phenotype [7, 9–12].

Monocytes and macrophages constitute one of the largest cell populations in solid tumors, including glioblastoma [13]. The recruitment of myeloid cells has been associated with poor responses to therapy, disease recurrence [13], and the development of acquired resistance to therapies, including anti-angiogenesis strategies [14–16]. Our group recently reported that Tie2-expressing monocytes (TEMs), a subpopulation of circulating blood monocytes that was previously identified as pro-angiogenic and immunosuppressive [17, 18], are overrepresented at the invasive front in surgical samples of human glioblastoma and murine glioma models after anti-VEGF therapy [12, 15]. Perivascular TEMs (MRC1^+^CXCR4^+^) have recently been associated with tumor relapse after chemotherapy in murine and human adenocarcinomas [19]. Our previous results further illustrated the role of TEMs in specifically enhancing the invasive properties of glioma cells by secreting high levels of gelatinase enzymatic proteins [15]. These findings provided evidence for a specific and targetable monocytic subpopulation associated with the heightened tumor invasion observed after anti-angiogenesis therapy.

Angiopoietins (Ang) are a family of molecules that bind to and regulate the Tie2 receptor [20, 21] and includes Ang1, Ang2, and Ang3 (Ang4, mouse ortholog). Meanwhile Ang1 is considered the main agonist Tie2 ligand, Ang2 effect on tumor growth is context-dependent [20]. In addition, Ang2 has emerged as a key molecule in inflammation [22]. The Ang1/Ang2 ratio has been correlated with the degree of vascular normalization and with the survival of patients with glioblastoma [23, 24].

Our aim in this study was to better understand the mechanisms that regulate the tumor microenvironment after anti-angiogenesis therapy for glioblastoma. We show the upregulation of Ang2 expression following anti-angiogenesis therapy in syngeneic and xenogeneic murine glioma models. We gathered evidence demonstrating that Ang2 attracts TEMs both *in vitro* and *in vivo* and enhances the tumor-remodeling properties of this specific monocyte subpopulation. We also show that exogenous soluble Tie2 expression significantly reduced TEM recruitment and, of clinical importance, completely abrogated the invasive phenotype induced by anti-angiogenesis therapy. These results illustrate the role of Ang2 in the acquired invasive properties of gliomas that result from targeting the VEGF pathway and the antagonistic role of soluble Tie2 in this process.

## RESULTS

### The invasive phenotype observed after anti-VEGF therapy is associated with increased Ang2 levels

**Figure 1:**
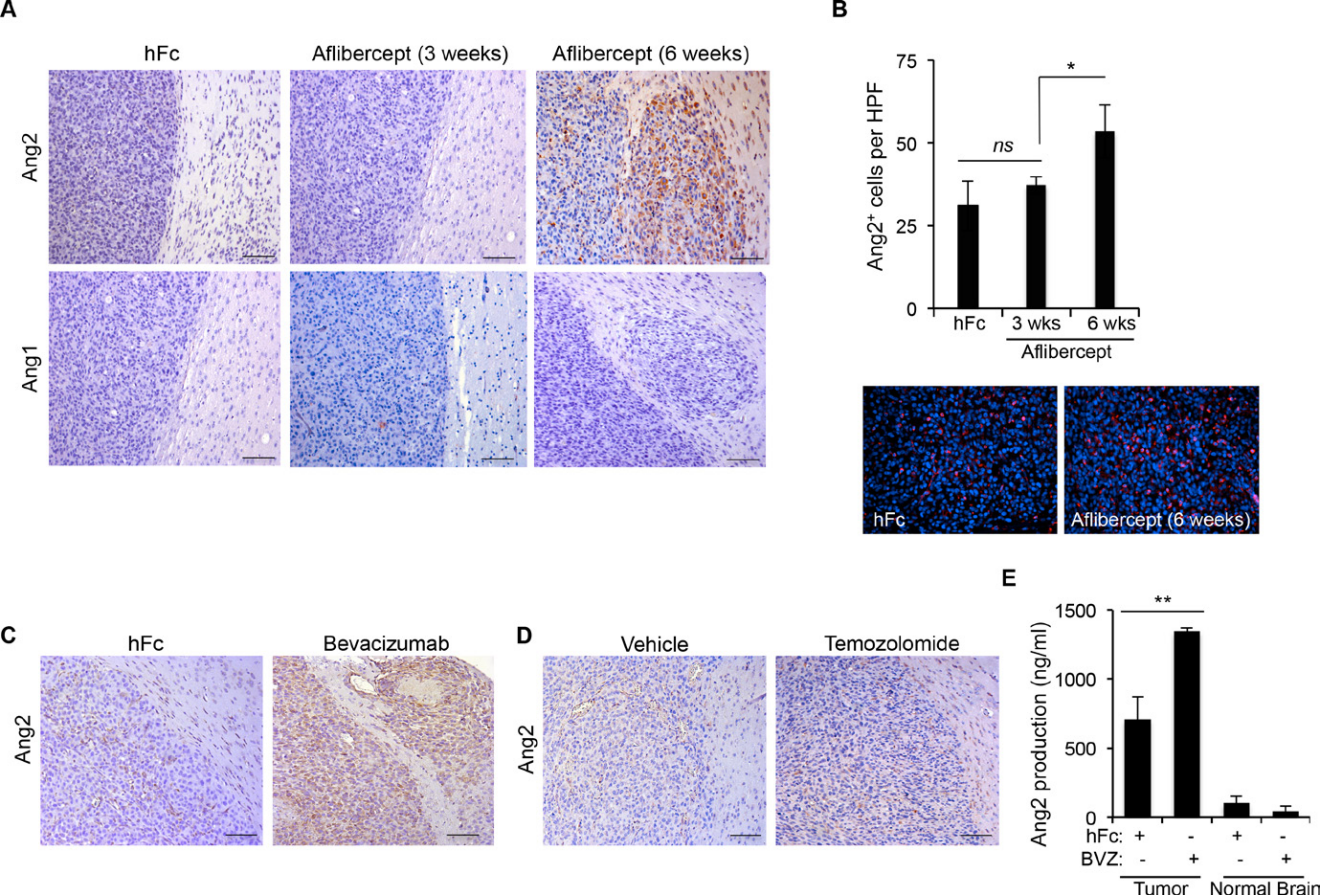
Anti-VEGF therapy-induced invasive tumor phenotype is associated with increased Ang2 expression (**A**) Sections of U87MG-derived tumors from mice treated with aflibercept for 3 weeks or 6 weeks or with control treatment (hFc) were stained for Ang2 and Ang1 expression. Invasive features and increased Ang2 were observed in animals treated with aflibercept for 6 weeks. Scale bars = 50 μm. (**B**) Quantification (top) of Ang2^+^ cells in tumors from animals treated with aflibercept (3 or 6 weeks) or control. Data are presented as mean ± SD. Representative images (bottom) show merged fluorescent Ang2 (red) and DAPI (blue). HPF, high-power field. ns, *P* > 0.05; *P < 0.05. (**C**, **D**) Tumor sections from mice treated with bevacizumab (C), temozolomide (D), or controls were stained for Ang2 expression. Scale bars = 50 μm. (**E**) Quantification by enzyme-linked immunosorbent assay of Ang2 production in tumor lysates from U87MG-derived intracranial xenografts after treatment with bevacizumab or control (hFc) compared with Ang2 present in normal brain tissue lysates. Data are presented as mean ± SD. BVZ, bevacizumab. **P < 0.01.

Our group previously reported the acquisition of an invasive phenotype and the overrepresentation of TEMs at areas of invasion in gliomas following anti-VEGF therapy [12, 15]. In addition, we showed that TEMs enhanced the invasive properties of glioma cells [12, 15]. Here, we assessed whether Tie2 main ligands, Ang1 and Ang2, were upregulated after anti-VEGF therapy in these tumors. Using brain tissue sections from U87MG glioma–bearing athymic mice treated with the anti-VEGF agent aflibercept or control, we performed immunostaining for Ang1 and Ang2. Of note, two schedules of aflibercept treatment were analyzed since previous studies showed that short treatment (3 weeks) did not enhance invasion or recruitment of TEMs, whereas long treatment (6 weeks) enhanced both invasion and recruitment [12, 15]. While Ang1 expression levels remained low after aflibercept treatment, Ang2 expression dramatically increased after the long treatment (associated to invasive pattern) but not after the short treatment (Figure 1A). Interestingly, the increased Ang2 expression was circumscribed mainly to the periphery of the tumor and to invasive nodules (Figure 1A), following the same localization pattern previously observed for TEMs [15]. Significantly more cells expressed Ang2 after the long aflibercept treatment than after the control treatment or the short treatment (Figure 1B).

We then sought to determine whether Ang2 also increased after other VEGF-targeting approaches. For this purpose, we obtained brain tissue sections from U87MG-bearing athymic mice treated with a control or the VEGF-targeting agent bevacizumab and performed immunohistochemical staining for Ang2. Supporting our previous observation, Ang2 increased dramatically after treatment with bevacizumab, and this increase was particularly noticeable in areas of invasion (Figure 1C). Interestingly, treatment with temozolomide —a DNA-alkylating agent that is used as standard therapy for patients with malignant gliomas that does not recruit TEMs or induce an invasive glioma phenotype [15]—did not increase Ang2 expression (Figure 1D). Our results were corroborated by the analysis of Ang2 protein levels from lysates obtained from U87MG glioma–bearing athymic mice treated with bevacizumab. Specifically, we performed enzyme-linked immunosorbent assays to quantify the concentrations of Ang2 in the tumor tissue and in normal brain tissue from the contralateral hemisphere. In agreement with the immunohistochemical data, the Ang2 concentration increased in lysates obtained from tumor-bearing hemispheres but not in normal hemispheres after treatment with bevacizumab (Figure 1E).

### Ang2 stimulates migration of TEMs *in vitro* and *in vivo*

**Figure 2:**
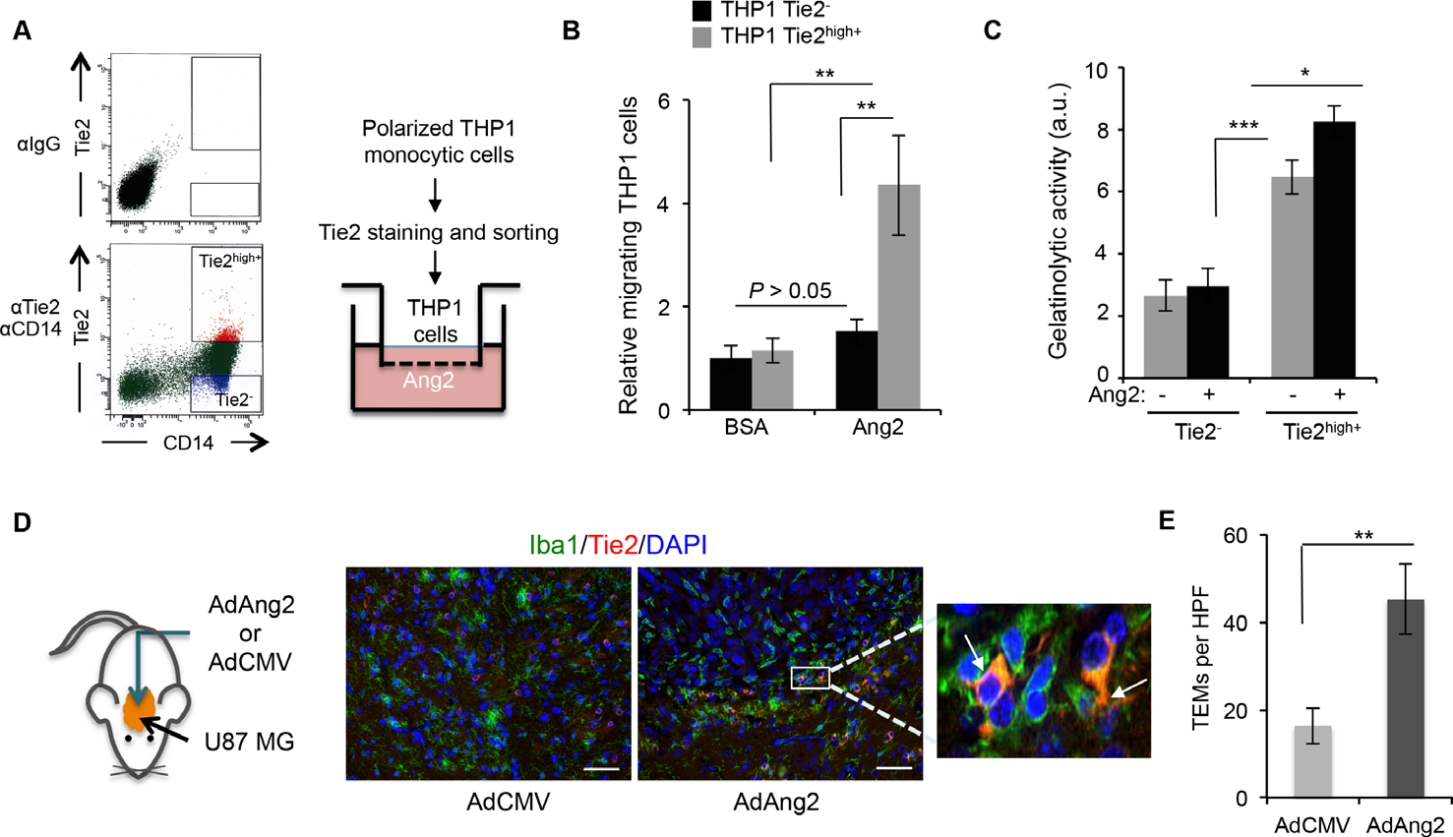
Ang2 is an attractant signal for TEMs *in vitro* and *in vivo* (**A**) THP-1 monocytic cells were exposed to IL-4, IL-13, and hypoxia and sorted as Tie2^−^ and Tie2^high+^ (left panel). Their migratory properties towards Ang2 or vehicle (BSA) were measured using a modified Boyden chamber as depicted (right panel). (**B**) Data from experiment described in (A) are presented as relative mean ± SD number of migrating THP-1 cells per microscopic field (200x). ns, *P* > 0.05; **P < 0.01. (**C**) Quantification of gelatinolytic activity present in conditioned medium, containing Ang2 or vehicle, of sorted CD14^+^Tie2^−^ and CD14^+^Tie2^high+^ cells. Data are presented as mean ± SD. au, arbitrary units. *P < 0.05; ***P < 0.001. (**D**) Brains of athymic mice bearing intracranial U87MG-derived tumors, treated with intratumoral delivery of an adenoviral vector expressing Ang2 (AdAng2) or a control vector (AdCMV), were analyzed for Iba1 (green) and Tie2 (red) expression. DAPI was used for nuclear staining (blue). White arrows indicate the presence of Iba1^+^Tie2^+^ cells. Scale bars = 20 μm. (**E**) Quantification of TEMs, defined as Iba1^+^Tie2^+^ cells, in surgical U87MG-derived xenografts treated intratumorally with AdCMV or AdAng2. Data are presented as mean ± SD. HPF, high-power field. **P < 0.01.

Since the increase in Ang2 expression and the recruitment of TEMs followed similar patterns, we hypothesized that Ang2 is responsible for the recruitment of TEMs to these tumors. To test this hypothesis, we performed migration assays of monocytic cells in the presence of Ang2. THP-1 monocytic cells were polarized, induced to express Tie2, and sorted into Tie2^−^ and Tie2^high+^ using previously published methods [15]. The migration of these populations towards Ang2 was then evaluated (Figure 2A). Our results showed significantly more migrating cells in the Tie2^high+^ THP-1 culture than in the Tie2^−^ counterparts (Figure 2B).

We previously demonstrated that a Tie2^high+^ cell population obtained from monocyte-enriched human peripheral blood mononuclear cells (PBMCs) exhibited enhanced tumor-remodeling activity compared with Tie2^−^ monocytic cells [15]. To understand the role of Ang2 in this activity, we collected conditioned media from Tie2^high+^ and Tie2^−^ cell populations that had been exposed to Ang2 or a vehicle, and performed a gelatinase activity assay. We observed that conditioned media from Tie2^high+^ populations displayed greater enzymatic activity than that from Tie2^−^ populations (Figure 2B), as we previously reported [15], and that this activity was enhanced in the presence of Ang2 (Figure 2C).

To evaluate whether these *in vitro* findings had any relevance in the *in vivo* setting, we proceeded to determine if increased Ang2 expression in the tumor resulted in higher number of recruited TEMs. For this purpose, we analyzed brain tissue sections from U87MG glioma–bearing mice treated with an adenovirus overexpressing Ang2 (AdAng2) and quantified the presence of TEMs by performing double immunofluorescence for Tie2 and Iba1 (Figure 2D). We observed significantly more Tie2^+^Iba1^+^ (i.e., double–positive) cells in tumors treated with AdAng2 than in tumors treated with a control vector (Figure 2D, 2E). Together, these results indicate that Ang2 is a chemoattractant signal for TEMs both *in vitro* and *in vivo*, suggesting that the increased Ang2 expression observed upon anti-angiogenesis therapy is at least partially responsible for the higher representation of TEMs in those tumors, enhancing the tumor-remodeling properties of this monocytic subpopulation.

### DC101 treatment increases Ang2 expression, TEMs recruitment, and invasive outgrowth in a syngeneic mouse model

**Figure 3:**
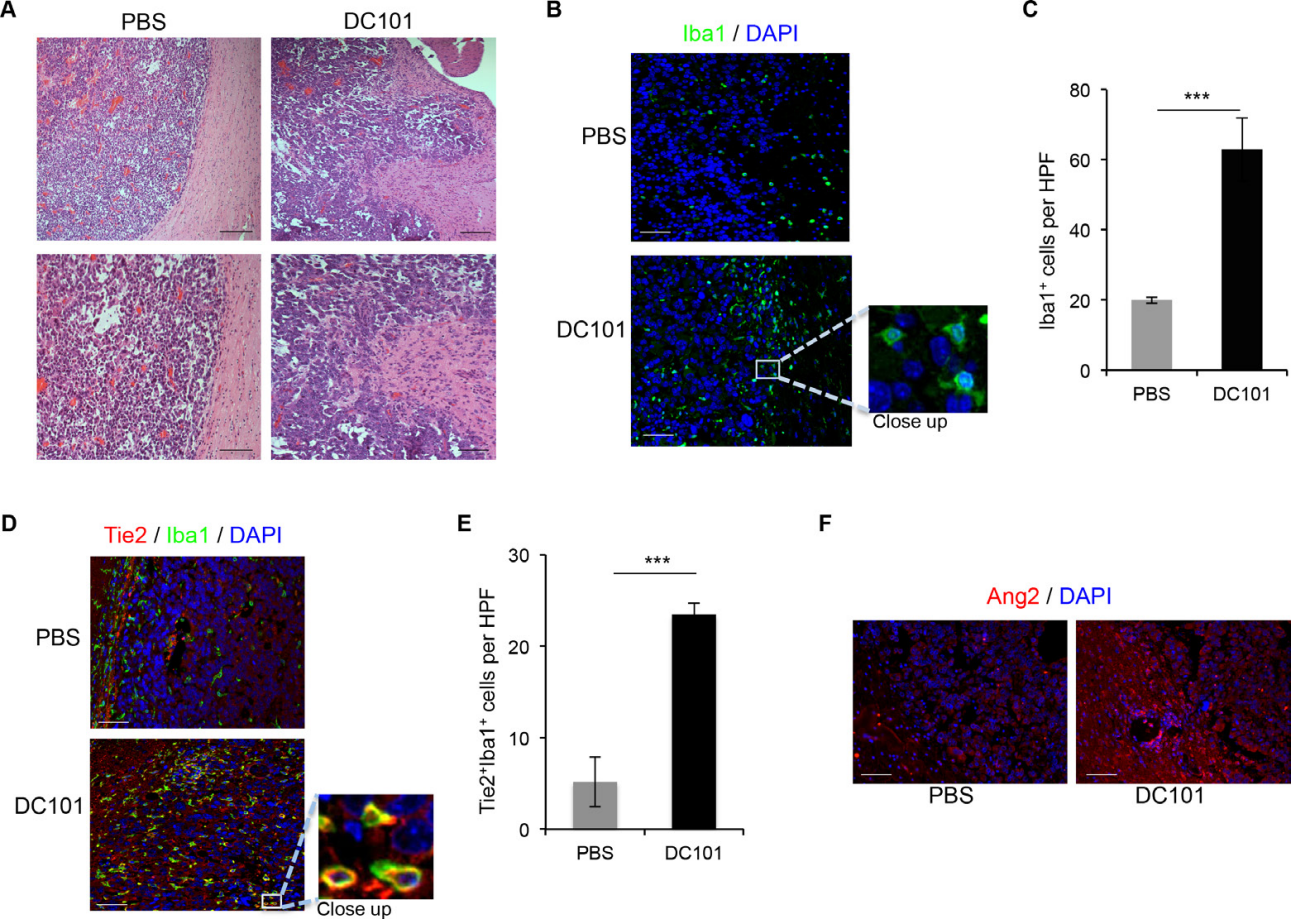
DC101 treatment increased Ang2 levels, TEM recruitment, and invasive glioma outgrowth (**A**) DC101 treatment induced invasive tumor outgrowth in the GL261 syngeneic model. Tumor sections from mice treated with DC101 or vehicle were stained with hematoxylin and eosin. Scale bars = 200 μm (top) and 50 μm (bottom). (**B**) Representative images of Iba1 immunostaining of brain sections of GL261-bearing mice treated with DC101 or vehicle. DAPI was used for nuclear staining (blue). Scale bars = 50 μm. (**C**) Quantification of Iba1^+^ cells present in a high-power field (HPF) in brain tumors after DC101 treatment. Data are presented as mean ± SD. ***P < 0.001. (**D**) Representative images of Tie2 (red) and Iba1 (green) double immunofluorescence in sections from GL261 syngeneic tumors treated with DC101 or vehicle. DAPI was used for nuclear staining (blue). Scale bars = 50 μm. (**E**) Quantification of TEMs (Tie2^+^Iba1^+^ cells) present in a HPF after DC101 treatment. Data are presented as mean ± SD. (**F**) Representative images of Ang2 immunofluorescence in sections from orthotopic GL261-derived gliomas after DC101 treatment. Scale bars = 50 μm. ***P < 0.001.

To validate our data, we tested the effect of anti-angiogenesis therapy in an immune-competent model. To this end, we implanted GL261 mouse glioma cells intracranially in C57BL/6 mice and treated them with DC101, a murine anti-VEGFR2 antibody [25]. We analyzed the histology of the resulting tumors and observed that GL261 generated well-limited gliomas, as expected [26]. However, in mice treated with DC101, the interface between the tumor and normal brain tissue was disrupted by finger-like tumor projections into the adjacent normal brain and invasive nodules (Figure 3A).

We then determined whether treatment with DC101 led to macrophage recruitment, as observed in immunodeficient models [15], by performing Iba1 staining of tissue sections from mice treated with DC101 or control treatment. Quantification of Iba1^+^ cells revealed a significant increase in macrophage recruitment in the periphery of the tumors from mice treated with DC101 compared with control-treated mice (Figure 3B, 3C). In addition, DC101 treatment resulted in overrepresentation of TEMs, identified by immunofluorescence as Iba1^+^Tie2^+^ cells when compared to control treatment group (Figure 3D, 3E). Finally, we detected increased Ang2 expression upon DC101 treatment versus control treatment (Figure 3F). Our data confirm that DC101 leads to changes in the tumor microenvironment in an immune-competent model similar to those caused by human anti-VEGF therapies. In addition, the established model was found to be suitable for studying the effect of targeting the Ang-Tie2 pathway in combination with VEGF-targeted approaches.

### Targeting the Ang-Tie2 axis abrogates the pro-invasive phenotype observed upon anti-angiogenesis therapy

**Figure 4:**
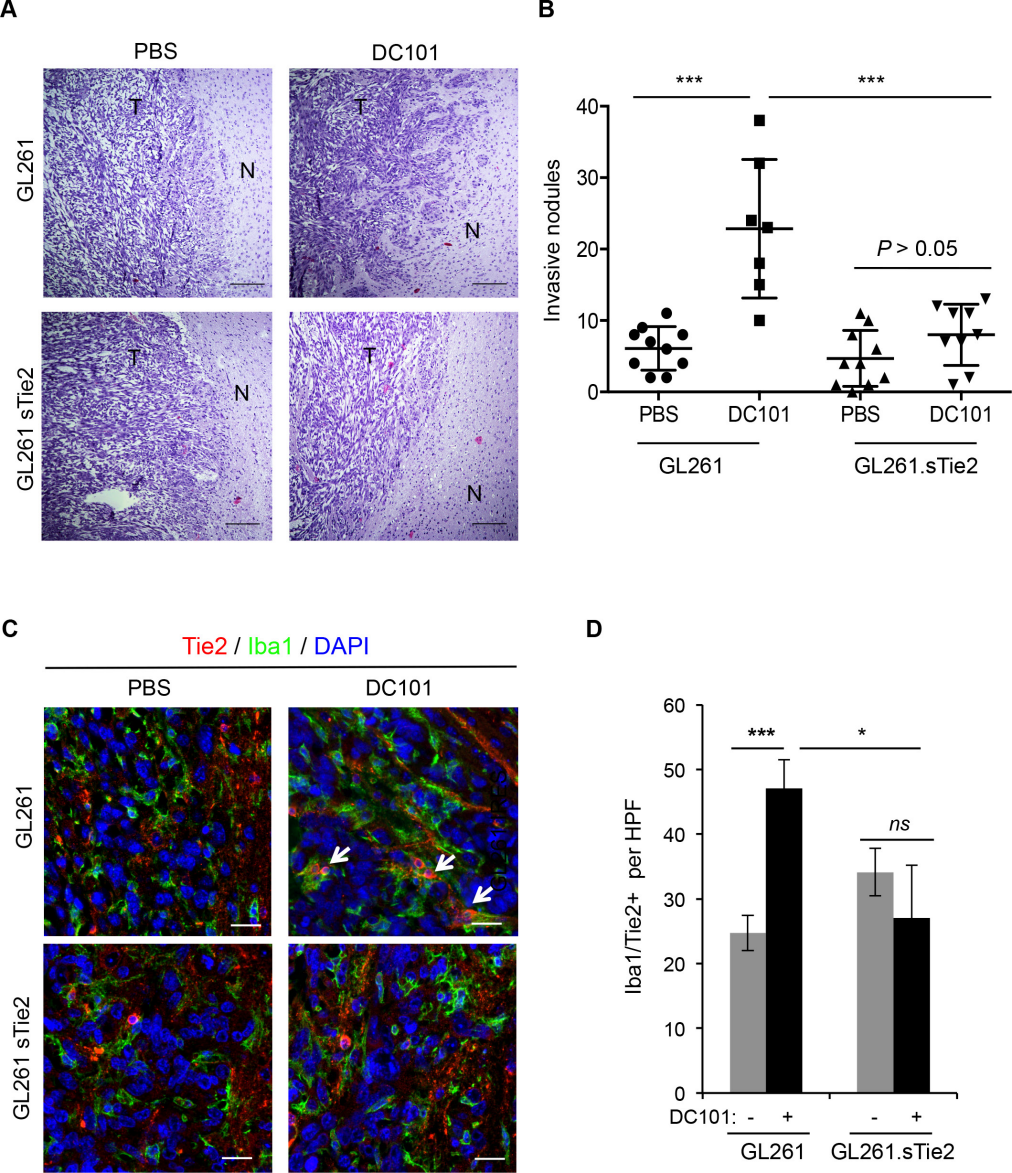
Soluble Tie2 expression counters the pro-invasive phenotype upon anti-VEGF therapy (**A**) GL261- and GL261. sTie2-derived tumor sections from mice treated with DC101 or vehicle were stained with hematoxylin and eosin. Invasive features were observed in Gl261-bearing mice treated with DC101 but not in GL261.sTie2bearing mice treated with DC101. Scale bars = 20 μm. N, normal brain; T, tumor. (**B**) Quantification of invasive nodules present in intracranial syngeneic tumors after DC101 treatment. GL261 and GL261.sTie2 were tested. Data are presented as mean ± SD. ***P < 0.001. (**C**) Representative images of Tie2 (red) and Iba1 (green) double immunofluorescence in sections from GL261 and GL261.sTie2 syngeneic tumors treated with DC101 or vehicle. DAPI was used for nuclear staining (blue). White arrows indicate the presence of Iba1^+^Tie2^+^ cells. Scale bars = 20 μm. (**D**) Quantification of TEMs (Tie2^+^Iba1^+^ cells) present in a high-power field (HPF) after DC101 treatment. Data are presented as mean ± SD. ns, *P* > 0.05; *P < 0.05; ***P < 0.001.

Because we showed that anti-angiogenesis therapy increases Ang2 expression and that Ang2 attracts TEMs, a monocytic subpopulation with tumor-remodeling characteristics, to the tumor/normal tissue interface, we then aimed to target the Ang2/Tie2 axis to counter the invasive phenotype observed after the use of anti-angiogenesis therapies. To this end, we implanted GL261.sTie2 (i.e., murine glioma cells overexpressing soluble Tie2 as a decoy receptor) [27] or control vector-transfected GL261 into C57BL/6 mice and treated these mice with DC101 or with a vehicle as a control. Tumors from vehicle-treated mice implanted with either GL261 or GL261.sTie2 were well circumscribed, with no evident signs of invasion (Figure 4A). As expected, tumors from DC101-treated GL261-bearing mice exhibited significantly more invasive nodules and finger-like projections into normal brain tissue than did those of vehicle-treated mice. Of interest, after DC101 treatment GL261.sTie2-derived tumors displayed markedly fewer invasive nodules than did GL261-derived tumors. Furthermore, DC101 treatment did not increase the number of invasive nodules in the GL261.sTie2-derived tumors (Figure 4B). Of note, when survival was analyzed, DC101 prolonged the survival of mice bearing either GL261 or GL261.sTie2-derived tumors (*P* < 0.001, log rank test); however, soluble Tie2 did not confer survival advantage in this model.

To evaluate TEM recruitment, double immunofluorescence for Iba1 and Tie2 was performed in tissue sections, and double-positive cells were identified and quantified. As expected, DC101 significantly increased the number of TEMs in GL261-derived tumors compared with those treated with a vehicle. However, GL261.sTie2-derived tumors failed to efficiently recruit TEMs after antiangiogenesis therapy (Figure 4C, 4D). Together, these results suggest that the expression of soluble Tie2 was sufficient to block TEM recruitment and prevent the appearance of an invasive phenotype after anti-angiogenesis therapy.

## DISCUSSION

**Figure 5:**
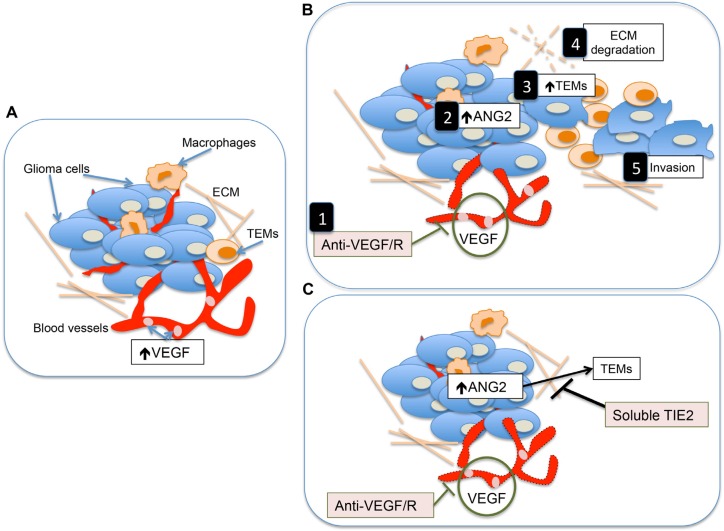
Proposed model describing the mechanisms underlying the heightened invasion of gliomas after anti-VEGF/R therapy and the role of soluble Tie2 in contrarresting this effect (**A**) Simplified representation of the tumor microenvironment in malignant gliomas. Two of the major characteristics of these tumors is the presence of high levels of VEGF and their highly angiogenic component. (**B**) Treatment with anti-VEGF or anti-VEGFR therapies results in not only an antiangiogenic effect on the tumor [1] but also in increased levels of Ang2 [2] which is a major attractant of TEMs [3]. This monocytic subpopulation is responsible for the production of tumor remodeling proteins and the subsequent breakdown of the extracelullar matrix (ECM) [4] favoring tumor invasion [5]. (**C**) The concomitant use of soluble Tie2 with antiangiogenesis therapies should, among other functions, jeopardize the effect of Ang2 to attract TEMs, preventing the induction of the highly invasive phenotype of gliomas associated with the use of anti-angiogenic therapies.

Understanding the mechanisms through which tumors acquire highly invasive and malignant behavior after anti-VEGF therapy has been the focus of extensive efforts from the scientific community [4, 9, 15, 16, 28, 29]. In this work, we provide evidence that the Ang2-Tie2 pathway is a key component in this process. We show that Ang2 generates a tumor microenvironment permissive of invasion through the recruitment of TEMs. In addition, concomitant targeting the Ang2-Tie2 pathway prevents the development of the invasive tumor phenotype observed after anti-VEGF therapy (Figure 5).

Inflammatory cell types regulate the adaptive response of malignant gliomas to anti-angiogenesis therapies [14–16]. Our group first described the overrepresentation of TEMs in surgical samples of human malignant gliomas after bevacizumab treatment and in preclinical xenograft models of glioma after bevacizumab and aflibercept treatment [15]. The presence of TEMs in the tumors was associated with the heightened invasive phenotype observed upon the use of anti-angiogenesis therapies [12, 15]. An increased percentage of TEMs has also been observed in the RIP1-Tag2 murine model of neuroendocrine tumors of the pancreas (PNET) after VEGFR inhibition [30]. Of interest, perivascular TEMs have recently been associated with tumor relapse after chemotherapy in murine and human adenocarcinomas [19]. Together these results suggest that TEMs might be associated with the recurrence and resistance of tumors to therapy including anti-angiogenesis strategies.

These data warranted investigating the chemoattractant signals responsible for the recruitment of this specific subpopulation of monocytes. To this end, we explored the expression of the two main ligands of Tie2, Ang1 and Ang2, and we observed an increase in Ang2 protein levels within the tumors treated with anti-VEGF or anti-VEGFR2 agents. Interestingly, the increased levels are not seen after anti-angiogenesis therapy administered using short schedule treatment (3 weeks) that did not result in an invasive tumor outgrowth, but after prolonged therapies (6 weeks) that were associated to heightened invasion. These findings agree with a clinical report on the correlation between high Ang2 levels and resistance to anti-VEGF therapy in glioblastoma patients [23]. Of interest, this relationship is not a universal phenomenon, as upregulation of Ang2 was observed in preclinical models of PNET in RIP-Tag2 mice but not in a model of MMTV-PyMT mammary carcinoma [30]. Ang2 has been shown to be a chemoattractant to TEMs *in vitro* [18] and in a syngeneic lung carcinoma model [31]. Our *in vivo* data revealing that Ang2 is responsible for attracting TEMs into murine gliomas, further support a role for Ang2 as chemoattractant molecule for TEMs observed after anti-angiogenesis therapies.

Although the mechanism by which Ang2 levels increase after anti-angiogenic therapies is not completely understood, several reports are describing VEGF as having a role in inducing Ang2 [32–34]. Interestingly, increased tissue and plasma VEGF levels are observed after anti-VEGF therapies (rev. in *[9, 35]*) that might be, in turn, responsible for Ang2 overexpression. Another putative mechanism might be related to the transcriptional regulation of Ang2 by Ets1 [36, 37], which is elevated in response to hypoxic conditions [38] that may be caused by anti-angiogenesis strategies [11].

Our group previously reported that TEMs exhibited greater secretion of matrix metalloproteinases compared with Tie2^−^ monocytes [15]. Here, we observed that Ang2 further enhanced the gelatinolytic properties of TEMs but not Tie2^−^ monocytes. Although Ang2 expression has been previously associated with invasion in gliomas, this invasion has not previously been linked to TEM recruitment but rather to mechanisms such as the induction of MMP2 expression via integrins [39]. Recent studies might be supporting the suggested interrelation among Ang2, TEMs, and invasion. Thus, blocking Ang2 resulted in limiting breast metastasis, at least in part by impeding TEM association with tumor blood vessels [40]. Furthermore, VEGFA signaling from TEMs modulated vascular junctions and tumor cell intravasation [41]. Our data indicate that TEM recruitment is at least one of the mechanisms underlying the enhanced invasion that accompanies Ang2 overexpression after anti-VEGF therapy.

While several studies have assessed the efficacy of targeting Ang2 in combination with anti-VEGF therapy [42], we tested the use of soluble Tie2 based on the rational that soluble Tie2 might also block the association between angiopoieitins and integrins previously associated with invasiveness in gliomas [39], and that other angiopoietins have been reported to have a role in glioma resistance to therapies [43]. In fact, a previous report described a role for soluble Tie2 in countering the development of tumor metastasis in murine models of breast cancer and melanoma [44]; however, the use of soluble Tie2 for treating cancer, including gliomas, has not yet addressed the impact of the treatment on the invasive phenotype induced by anti-angiogenesis therapy [27]. Our results show that inhibiting the Ang2-Tie2 pathway via soluble Tie2 prevented the recruitment of TEMs to the tumor and, of great interest, jeopardized the heightened invasive phonotype induced by anti-angiogenesis therapy. We did not observe an association between the soluble Tie2-mediated decrease in invasion and prolonged survival, suggesting that the heightened invasion occurring after anti-angiogenic therapies is not the only escape mechanism of gliomas to this treatment.

Although further studies to validate our results are warranted, the observed effect of soluble Tie2 on the invasive pattern of glioma progression induced by anti-angiogenesis therapy prompts us to envision the possibility of using this strategy to render recurrent gliomas more surgically suitable and in turn improve patient survival. In summary, our results illustrate mechanisms of brain tumor dispersal and encourage the testing of anti-angiogenesis therapies together with strategies that mimic the functions of a decoy Tie2 receptor.

## MATERIALS AND METHODS

### Cell lines and culture

U87MG human glioma cells (ATCC, Manassas, VA) were cultured in Eagle's minimum essential medium with 10% fetal bovine serum (FBS) and 1% essential amino acids. THP-1 monocytic leukemia cell line (ATCC) was cultured in RPMI 1640 medium supplemented with 2 mM L-glutamine and 10% FBS. GL261 mouse glioma cells were obtained through the tumor repository at the National Cancer Institute at Frederick National Laboratory for Cancer Research (Frederick, MD). GL261 pHR-Control and GL261 pHR-sTie2 were a kind gift from Dr. Luc Vallières (Centre Hospitalier de l'Université Laval, Quèbec, CA) [27]. GL261 cells were cultured in Dulbecco's Modified Eagle Medium/F12 supplemented with 10% FBS.

### Adenoviral vectors

The generation of the adenoviral vector expressing Ang2 (AdAng2) and of the control vector (AdCMV), containing an empty expression cassette, was described previously [45].

### Animal experiments

U87MG cells (5 × 10^5^) were implanted intracranially in the caudate nucleus of athymic mice, and the mice were treated as described before [12]. Briefly, aflibercept was administered subcutaneously at 25 mg/kg twice per week starting 10 days after implantation and continuing for 3 weeks or 6 weeks. Bevacizumab was administered intraperitoneally at 10 mg/kg starting on day 5 after implantation and continuing for 6 weeks. Temozolomide was administered intraperitoneally at 7.5 mg/kg on days 4–8 and 11–22 after implantation.

To assess the effect of Ang2 *in vivo*, U87MG cells (5 × 10^5^) were implanted in athymic mouse brains, and AdCMV or AdAng2 was administered intratumorally (1.5 × 10^8^ plaque-forming units) twice per week for 3 weeks.

GL261, GL261 pHR-Control, or GL261 pHR-sTie2 mouse glioma cells (2 × 10^5^) were implanted intracranially into the caudate nucleus of C57BL/6 mice as described before [26]. DC101 was administered intraperitoneally at 800 μg/dose three times per week for 4 weeks starting 3 days after implantation. Animals exhibiting generalized or localized signs of disease were sacrificed. Brains were extracted, formalin fixed, and paraffin embedded. All animal studies were performed in the veterinary facilities of MD Anderson Cancer Center in accordance with the institutional guidelines. Hematoxylin-and-eosin-stained sections were evaluated tumor growth and invasion. Quantification studies were performed with 3–5 mice per group.

### Immunohistochemical analysis

Five-micrometer sections of tissue were deparaffinized in three washes of xylene for 5 minutes each, followed by rehydration in serial dilutions of ethanol as follows: 100% ethanol twice for 5 minutes, 95% ethanol twice for 5 minutes, and 70% ethanol once for 5 minutes. Endogenous peroxidase activity was quenched using 3% H_2_O_2_ in 100% methanol for 10 minutes at room temperature. Heat-induced antigen retrieval was performed in a pre-warmed solution of 10 mM citrate buffer (pH 6.0) for 10 minutes in a pressure cooker. Slides were allowed to cool down at room temperature for 30 minutes before they were washed and blocked using a filtered 5% bovine serum albumin (BSA) in phosphate-buffered saline (PBS) solution. Primary antibodies were used: Ang2 (IHC World, Woodstock, MD) and Ang1 (1:100; R & D Systems, Minneapolis, MN; diluted using IHC-Tek diluent (pH 7.4; IHC World) After a 4°C overnight incubation with primary antibodies, biotin-conjugated secondary antibodies (Vector Laboratories, Burlingame, CA) were diluted 1:500 in IHC-Tek diluent and applied to tissue sections for 1 hour at room temperature. This was followed by peroxidase-conjugated avidin (Elite ABC Kit, Vector Laboratories) incubation for 1 hour at room temperature. 3, 3′ Diaminobenzidine (Sigma-Aldrich, St. Louis, MO) was used for signal detection. Nuclei counterstaining was performed with hematoxylin, and slides were mounted with Cytoseal 60 (Thermo Scientific, Waltham, MA). Images were captured using a bright-field microscope (Axioskop 40; Zeiss, Jena, Germany).

### Immunofluorescence

Deparaffinization and rehydration, followed by heat-induced antigen retrieval and blocking, were performed as explained above. Primary antibodies were diluted using IHC-Tek diluent (pH 7.4; IHC World) as follows: Iba1 (EMD Millipore, Billerica, MA, 1:1000), Tie2 (C-20; Santa Cruz Biotechnology, Dallas, TX, 1:100), and Ang2 (IHC World, ready-to-use). After a 4°C overnight incubation, biotin-conjugated secondary antibodies were diluted 1:500 in IHC-Tek diluent and placed on tissue sections for 1 hour at room temperature. DyLight-conjugated streptavidin (1:1000; Vector Laboratories) was used for signal detection. When required, the signal was amplified by an additional step of biotin-conjugated anti-streptavidin antibody (1:1000; Vector Laboratories) for 30 minutes at room temperature followed by DyLight-conjugated streptavidin (1:1000; Vector Laboratories) for 30 minutes at room temperature. 4′, 6-Diamidino-2-phenylindole (DAPI) was applied for 15 minutes at room temperature for nuclear counterstaining. Slides were then mounted using Dako Fluorescence Mounting Medium (Carpinteria, CA). Ten images from each slide were taken using a Zeiss Axiovert 200 M microscope under a 20x objective and were manually quantified.

### Isolation of monocytes from peripheral blood of healthy donors

Peripheral blood mononuclear cells (PBMCs) were prepared from healthy donor blood (Gulf Coast Regional Blood Center, Houston, TX) by centrifugation on a Ficoll-Paque Plus (GE Healthcare, PA) density gradient at 950 *g* for 20 minutes with deceleration set at zero, as previously described [15]. PBMCs were incubated in the presence of 20 μl per 1 × 10^7^ cells of CD14 MicroBeads (Miltenyi Biotec) for 20 minutes at 4°C, after which cells were run through a magnetic column to isolate CD14-positive monocytes. The monocyte-enriched population was then double-stained with anti-human CD14-APC (R & D Systems) and anti-human Tie2-PE (R & D Systems) (both dilution 1:10) in the presence of cell-surface Fc receptor–blocking reagent (Miltenyi Biotec). The stained cell suspension was then subjected to flow cytometric analysis and cell sorting (FACSAria II; Becton Dickinson, Franklin Lakes, NJ) to obtain Tie2-negative (CD14^+^/Tie2^−^) and Tie2-expressing monocytes (CD14^+^/Tie2^high+^).

### Migration assay

For the migration assay, Tie2^high+^ and Tie2^−^ THP-1 cells were isolated as previously reported [15]. Briefly, THP-1 cells were placed under hypoxic conditions (1% O_2_, 5% CO_2_, and 94% N_2_) in the presence or absence of 20 ng/ml IL-4 and 20 ng/ml IL-13 (R & D Systems) for 48 hours. Then, cells were stained using phycoerythrin-conjugated anti-Tie2 antibody (1:50; R & D Systems) and subjected to flow cytometric analysis and cell sorting using Becton Dickinson FACSAria II. A total of 1 × 10^5^ sorted Tie2^high+^ and Tie2^−^ THP-1 monocytic cells were placed in transwells containing a 5-μm pore polycarbonate membrane (Corning, Corning, NY), which were then placed over a well containing 500 μl of Ang1 or Ang2 (400 ng/ml) (R & D Systems) in serum-free media or the equivalent amount of BSA. Cells were allowed to migrate for 24 hours, after which the number of migrated cells in the lower chamber was counted with a hemacytometer using Trypan Blue.

### Gelatinase assay

Gelatinolytic activity in conditioned medium from sorted Tie2^high+^ and Tie2^−^ THP-1 monocytic cells obtained from PBMCs was measured using an ENZChek Gelatinase/Collagenase Assay Kit (Invitrogen, Carlsbad, CA) according to the manufacturer's instructions and as previously reported [15].

### Enzyme-linked immunosorbent assay

To measure *in vivo* Ang2 production upon anti-VEGF therapy, brain tissue from U87MG-bearing mice treated with bevacizumab was compared with that from U87MG-bearing mice treated with vehicle. Tumor samples were homogenized in RIPA buffer (1% NP-40, 0.5% sodium deoxycholate, 0.1% sodium dodecyl sulfate, 50 mM Tris-Cl pH 7.5, 150 mM NaCl, 1X Protease Inhibitor Cocktail, 1X Phosphatase Inhibitor Cocktail) using a disposable pestle. After keeping the lysate on ice for 30 minutes, the lysate was centrifuged (13K, 10 minutes, 4°C), and the resulting supernatant was analyzed for Ang2 concentration using a Quantikine ELISA Kit (R & D Systems) per the manufacturer's instructions.

### Statistical analyses

For quantitative data analysis, means and standard deviations (SDs) were used. Statistical analyses were performed using GraphPad Prism version 6.0 (GraphPad Software, La Jolla, CA). The statistical significance of differences between groups was determined using the Student *t*-test. Survival studies were analyzed by the log rank test. *P* < 0.05 was considered statistically significant.
